# The role of extracellular vesicles in cholangiocarcinoma tumor microenvironment

**DOI:** 10.3389/fphar.2023.1336685

**Published:** 2024-01-10

**Authors:** Nuoqi Zhang, Lizhuang Shu, Zengli Liu, Anda Shi, Liming Zhao, Shaohui Huang, Guoli Sheng, Zhangdi Yan, Yan Song, Fan Huang, Yongchang Tang, Zongli Zhang

**Affiliations:** ^1^ Department of General Surgery, Qilu Hospital, Shandong University, Jinan, Shandong, China; ^2^ Department of General Surgery, Qilu Hospital, Shandong University, Qingdao, Shandong, China

**Keywords:** cholangiocarcinoma, tumor microenvironment, extracellular vesicles, exosome, EV-based therapy

## Abstract

Cholangiocarcinoma (CCA) is a highly aggressive malignant tumor that originates from the biliary system. With restricted treatment options at hand, the challenging aspect of early CCA diagnosis leads to a bleak prognosis. Besides the intrinsic characteristics of tumor cells, the generation and progression of CCA are profoundly influenced by the tumor microenvironment, which engages in intricate interactions with cholangiocarcinoma cells. Of notable significance is the role of extracellular vesicles as key carriers in enabling communication between cancer cells and the tumor microenvironment. This review aims to provide a comprehensive overview of current research examining the interplay between extracellular vesicles and the tumor microenvironment in the context of CCA. Specifically, we will emphasize the significant contributions of extracellular vesicles in molding the CCA microenvironment and explore their potential applications in the diagnosis, prognosis assessment, and therapeutic strategies for this aggressive malignancy.

## 1 Introduction

Cholangiocarcinoma (CCA) is the predominant form of primary malignant tumor originating from the biliary system (Blechacz et al., 2011). It can be categorized into three subtypes based on the anatomical site involved: intrahepatic cholangiocarcinoma (iCCA), perihilar cholangiocarcinoma (pCCA), and distal cholangiocarcinoma (dCCA). The worldwide occurrence of CCA has exhibited a consistent rise over the past four decades (Banales et al., 2020). CCA is highly invasive, and its clinical symptoms in the early stages are often subtle, leading to diagnostic challenges. Most patients receive a diagnosis when the disease has already progressed to an advanced stage. Surgical resection, often seen as the sole potentially curative treatment, is a viable option for around 20%–30% of patients. When combined with adjuvant capecitabine, resection can lead to a reported median survival of 53 months. Regrettably, for the remaining 70%–80% of patients with locally unresectable or distant metastatic disease, survival tends to be restricted to approximately 1 year (Moris et al., 2023).

**FIGURE 1:**
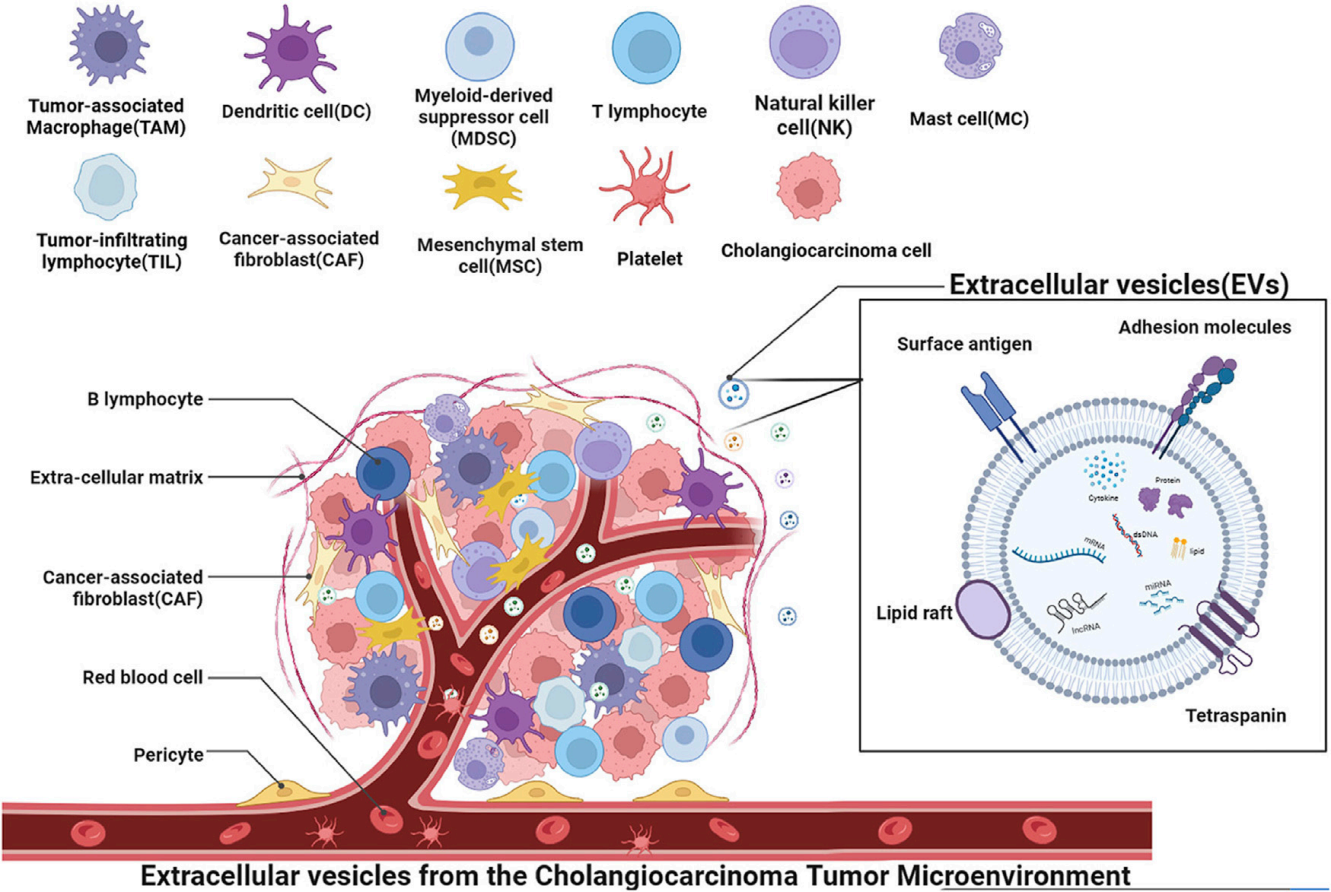
Schematic depiction of the role of extracellular vesicles in the context of cholangiocarcinoma microenvironment cells. The microenvironment is comprised of cancer cells, mesenchymal stem cells, cancer-associated fibroblasts, immune cells such as tumor-associated macrophages (TAM), mast cells (MC), natural killer cells (NK), tumor-infiltrating lymphocytes (TIL), vascular endothelial cells, and platelets associated with angiogenesis. Various cells interact through vesicles to form the cholangiocarcinoma tumor microenvironment, promoting tumor invasion, regulating tumor growth, promoting angiogenesis, and performing other functions. Extracellular vesicles, as a mode of transportation, can transfer various substances such as mRNA, lncRNA, miRNA, dsDNA, signaling proteins, lipids, cytokines, and other extracellular vesicles to target cells. These substances can regulate key processes in tumor progression.

Cholangiocarcinoma (CCA) is a desmoplastic hyperplastic tumor characterized by a unique tumor microenvironment (TME) (Wang and Ilyas, 2021). Beyond the influence of tumor cell epigenetics, tumor formation and advancement are primarily governed by the TME (Baghban et al., 2020). Reprogramming of tumor initiation, growth, invasion, metastasis, and the response to therapy heavily relies on the crucial role played by the TME (Jin and Jin, 2020). The interplay between tumor cells and the TME is a bidirectional, ever-changing process, involving both cell-cell and cell-free contact through various signaling molecules and extracellular vesicles (Figure 1). These secreted soluble molecules, cytokines, and extracellular vesicles are responsible for the transfer of genetic information horizontally among cells, facilitating cell-to-cell communication (Baghban et al., 2020). Among these, extracellular vesicles stand out as crucial carriers in mediating intercellular communication between tumor cells and the TME (Lemoinne et al., 2014).

Extracellular vesicles (EVs) are lipid bilayer spheres that encapsulate signaling proteins, lipids, nucleic acids (DNA and RNA), and metabolites, and they are released by diverse cell types into the extracellular media of various biological fluids, including serum, urine, bile, and saliva (Kahlert et al., 2014; Melo et al., 2015). Extracellular vesicles have the capacity to be conveyed to nearby or remote cells via a range of mechanisms, including direct interaction with the cellular membrane, fusion, or internalization (Colombo et al., 2014). These EVs carry signals to recipient cells and participate in intercellular communication in both physiological and pathological conditions. EVs are categorized based on their biogenesis into exosomes, microvesicles (MVs) or microparticles, and apoptotic bodies. Exosomes, which are produced within the multivesicular endosomes (MVEs) of cells, exhibit a spherical morphology and a diameter ranging from 40 to 150 nm. MVEs, generated during the maturation process of early endosomes, give rise to intraluminal vesicles (ILVs) through the invagination of the MVE membrane. Upon fusion of MVEs with the cell’s plasma membrane, ILVs are released into the extracellular media as exosomes. In contrast, MVs or microparticles originate from the direct budding of the cell’s plasma membrane and display heterogeneous sizes (ranging from 40 to 1,000 nm) and morphologies (Raposo and Stoorvogel, 2013). Apoptotic bodies, on the other hand, are vesicles produced by cells undergoing apoptosis, characterized by diverse sizes (∼40 to 2000–5,000 nm) and morphologies (Akers et al., 2013). EVs possess the remarkable ability to be transported to neighboring or distant cells through various mechanisms, including direct interaction with the cellular membrane, fusion, or internalization. Among these vesicles, exosomes have garnered greater research attention in this review due to their heightened significance. Bioactive substances carried by EVs exert regulatory control over crucial processes in tumor progression, such as inflammation promotion (Haga et al., 2015), reconfiguration of the cellular-matrix interface (Mu et al., 2013), angiogenesis (Bouvy et al., 2014), chemotherapeutic drug resistance (Chen et al., 2014; Shao et al., 2015), as well as suppression of anti-tumor immune responses (Chalmin et al., 2010). This paper delves into the intricate interactions among different components within the cholangiocarcinoma microenvironment, with a particular emphasis on elucidating the contribution of EVs.

## 2 The role of contents carried by extracellular vesicles in tumor microenvironment

**FIGURE 2:**
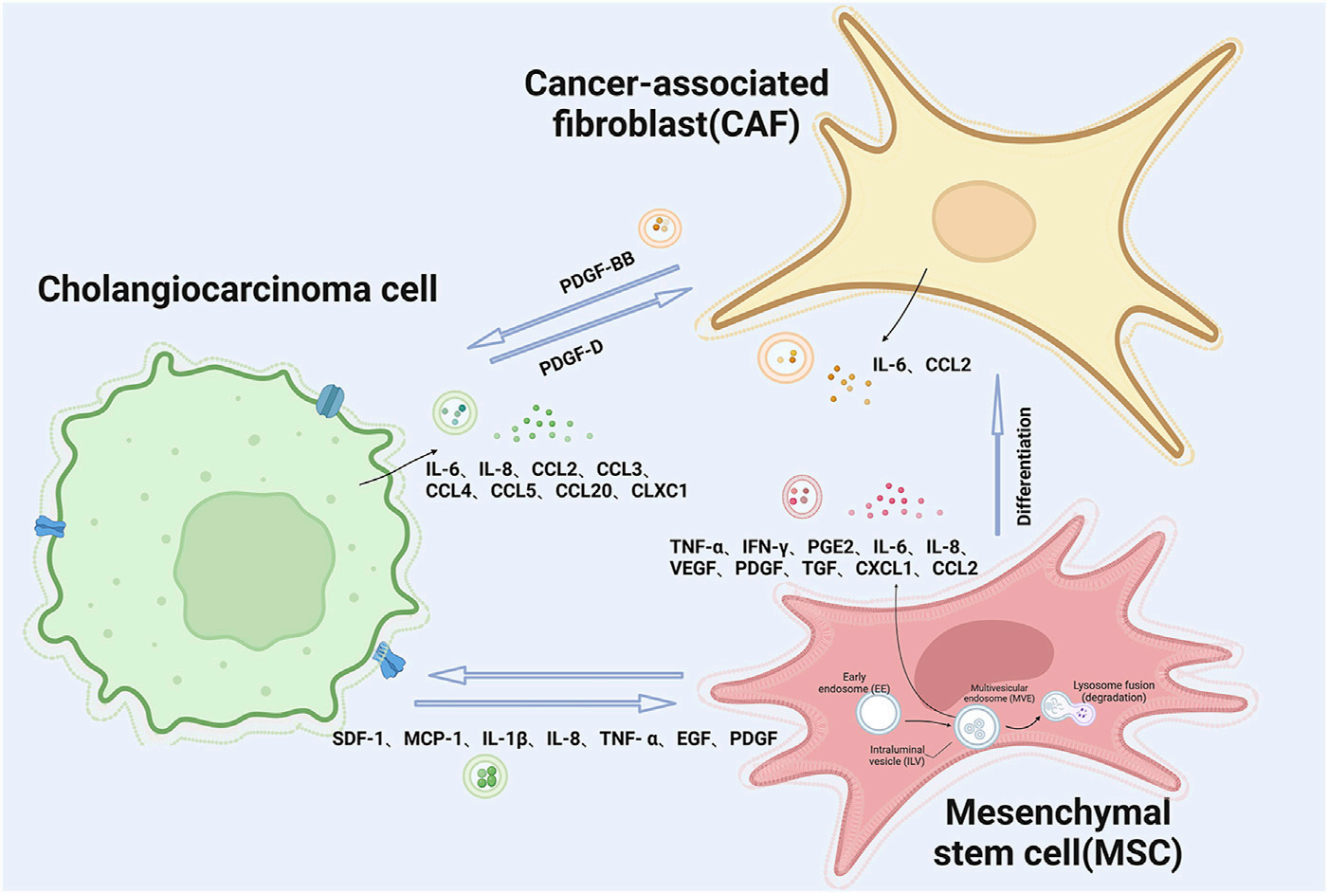
Schematic illustration of extracellular vesicle interactions between cholangiocarcinoma tumor cells and mesenchymal stem cells and cancer-associated fibroblasts.

Extracellular vesicles (EVs) serve as vital mediators of intercellular communication within TME (Lindoso et al., 2017), and they exhibit a remarkable degree of heterogeneity and dynamism, with their content, size, and membrane composition being contingent upon factors Including factors like the cellular source, its current state, and the surrounding environmental context (Yáñez-Mó et al., 2015). In this context, EVs assume a central role in coordinating the exchange of intercellular information, as they fuse with target cells to deliver a spectrum of contents (Figure 2), including proteins, lipids, mRNA, microRNAs, long non-coding RNAs (lncRNA), double-stranded DNA (dsDNA), and various other bioactive molecules (Raposo and Stoorvogel, 2013).

In CCA and its TME, EVs secreted by CCA cells stimulate cholangiocyte proliferation and promote their invasive behavior, and these events are related to the enrichment of oncoproteins in EVs. Proteins with varying expression patterns that are implicated in the progression of CCA encompass epidermal growth factor receptor (EGFR), Mucin-1, and Integrin β 4 (ITGB4), epithelial cell adhesion molecule (EPCAM) (Arbelaiz et al., 2017), Galactose Lectin 3 binding protein (LG3BP), Prostaglandin F2 receptor negative regulator (PTGFRN) and 4F2 cell surface atigen heavy chain (4F2hc) (Dutta et al., 2015), etc. EGFR contributes to tumor cell dedifferentiation and invasiveness, serving as an unfavorable prognostic indicator. Meanwhile, the upregulation of Mucin-1 and EPCAM in CCA is linked to an unfavorable prognosis among CCA patients (Clapéron et al., 2014). ITGB4 has been identified as an EV-associated integrin that plays a pivotal role in dictating the future metastatic site, thereby contributing to the selective organ-specific alignment of tumor cells (Hoshino et al., 2015). The serum exosomes of CCA are enriched in several proteins, such as aminopeptidase N (APN), pantothenate (VNN1), and polyimmunoglobulin receptor (PIGR) (Arbelaiz et al., 2017). Compared with primary sclerosing cholangitis (PSC) samples, fibrinogen gamma chain (FGG), α-1-acidic glycoprotein 1 (A1AG1), and S100A8 were stable in the EV of CCA samples (Sirica et al., 2019). These investigations suggest that the proteins found in exosomes have the potential to be regarded as early diagnostic indicators for CCA. Proteins transported by EVs as carriers in the tumor microenvironment of CCA are summarized in Table 1. Exosomes are capable of transporting a variety of bioactive lipids, including sphingomyelin, cholesterol, lysophosphatidylcholine, arachidonic acid, various fatty acids, prostaglandins, and leukotrienes, to diverse cellular destinations (Record et al., 2014). Research has shown that vesicle-bound lysophosphatidylcholine (Table 2) supports DC maturation and lymphocyte chemotaxis via G protein-coupled receptors (Subra et al., 2007). Additionally, vesicle-bound prostaglandins (Table 2) activate intracellular signaling pathways, such as PGE2, promoting immunosuppression for tumor development (Subra et al., 2010). Prior research has provided evidence that sphingomyelin (Table 2) primarily mediates the angiogenic activity of tumor-derived EVs *in vitro* and *in vivo* (Kim et al., 2002). However, while lipomics and exploring the complete lipid profile of EVs are emerging research areas, current lipid separation and analysis technologies limit the description of only a few lipid groups.

**TABLE 1:** Proteins of extracellular vesicles specifically expressed in the tumor microenvironment of cholangiocarcinoma.

Name	Source	Function	References
MUC-1/CD227	Cholangiocarcinoma cell	Promote the occurrence and development of tumor	Arbelaiz et al. (2017)
EGFR	Cholangiocarcinoma cell	Regulate tumor growth, enhance tumor malignancy, promote tumor infiltration and invasion	Arbelaiz et al. (2017)
ITGB4/CD104	Cholangiocarcinoma cell	Promote tumor development, infiltration and invasion	Arbelaiz et al. (2017)
EPCAM/CD326	Cholangiocarcinoma cell	Promote the occurrence and development of tumor	Arbelaiz et al. (2017)
LG3BP/M2BP	Cholangiocarcinoma cell	Promote the occurrence and development of tumor	Dutta et al. (2015)
SLC3A2/4F2 HC/CD98	Cholangiocarcinoma cell	Promote the occurrence and development of tumor	Dutta et al. (2015)
PTGFRN	Cholangiocarcinoma cell	Promote the occurrence and development of tumor	Dutta et al. (2015)
APN/CD13	Serum	Biomarkers for cholangiocarcinoma	Arbelaiz et al. (2017)
VNN1	Serum	Potential Biomarkers for cholangiocarcinoma	Arbelaiz et al. (2017)
PIGR	Serum	Potential Biomarkers for cholangiocarcinoma	Arbelaiz et al. (2017)
FGG	Serum	Potential Biomarkers for cholangiocarcinoma	Arbelaiz et al. (2017)
A1AG1	Serum	Potential Biomarkers for cholangiocarcinoma	Arbelaiz et al. (2017)
S100 A8	Serum	Potential Biomarkers for cholangiocarcinoma	Arbelaiz et al. (2017)
BMI 1	Cholangiocarcinoma cell	Regulate tumor growth and promote tumor infiltration and invasion	Liu et al. (2022)
MMP1	CAF, PLT	Regulate tumor growth and promote tumor infiltration and invasion	Nakagawa et al. (2004)
MMP9	Mast cell	Modulate inflammatory response	Komi and Redegeld (2020)
MT1-MMP/MMP14	Cholangiocarcinoma cell, CAF, PLT	Regulate tumor growth and promote tumor infiltration and invasion	Janowska-Wieczorek et al. (2005)
HSP 70	Cholangiocarcinoma cell	Exosome non-specific protein	Kim et al. (2006)
LFA-1	Mast cell, MSC	Induce tumor immune response	Xie et al. (2010)
ICAM-1/CD54	Mast cell, MSC, DC, Cholangiocarcinoma cell	Induce tumor immune response	Carroll-Portillo et al. (2012)
BSG/CD147/EMMPRIN	Cholangiocarcinoma cell, PLT	Promote tumor invasion, angiogenesis, support epithelial-mesenchymal transition (EMT)	Millimaggi et al. (2007)
APO 2L/TRAIL	Activated T cells	Induce apoptosis	Alonso et al. (2011)
MHC II	Mast cell, Activated T cells	Induce tumor immune response	Carroll-Portillo et al. (2012)
HSP	Mast cell	Induce tumor immune response	Carroll-Portillo et al. (2012)
FcεRI	Mast cell	Induce tumor immune response	Carroll-Portillo et al. (2012)
TCR	Activated T cells	Inhibition of anti-tumor immune response	Alonso et al. (2011)
Fas L	Activated T cells	Inhibition of anti-tumor immune response	Alonso et al. (2011)
NKG2DL	Activated T cells	Inhibition of anti-tumor immune response	Alonso et al. (2011)

**TABLE 2:** Lipid of extracellular vesicles specifically expressed in the tumor microenvironment.

Name	Source	Function	References
sphingomyelin	Cholangiocarcinoma cell	Promote angiogenesis	Kim et al. (2002)
lysophosphatidylcholine	Mast cell	Induction of DC maturation, chemotaxis of lymphocytes	Subra et al. (2007)
prostaglandin	Mast cell	Trigger prostaglandin-dependent intracellular signaling pathways in target cells	Subra et al. (2010)

The extracellular vesicles contain intact mRNA (Zuo et al., 2020), mRNA fragments (Lu et al., 2017), long non-coding RNAs(Liechty et al., 2000), microRNA (miRNA) (De Bruyn et al., 2011), etc. In certain cellular contexts, miRNA can be transported to neighboring cells via EVs, consequently modulating the gene expression and phenotypic traits of the receiving cells. RNAs transported by EVs in the CCA tumor microenvironment mentioned in this article is summarized in Table 3.

**TABLE 3:** RNAs of extracellular vesicles specifically expressed in the tumor microenvironment.

Name	Source	Function	References
miR-493-5p	CAF	Inhibit the anti-tumor immune response and enhance the malignant degree of tumor	Toshida et al. (2023)
miR-9-5p	Cholangiocarcinoma cell	Enhance tumor malignancy	Zhang et al. (2020)
miR-183-5p	Cholangiocarcinoma cell	Regulate tumor growth, promote tumor invasion, angiogenesis, support epithelial mesenchymal transition (EMT), and inhibit anti-tumor immune response	Shu et al. (2023)
miR-182-5p	Cholangiocarcinoma cell	Regulate tumor growth, promote tumor invasion, angiogenesis, support epithelial mesenchymal transition (EMT)	Shu et al. (2023)
miR-195	Cholangiocarcinoma cell, CAF	Inhibit angiogenesis	Xie et al. (2019)
miR-30e	Cholangiocarcinoma cell	Inhibit epithelial-mesenchymal transition, tumor invasion and angiogenesis	Ota et al. (2018)
circ-CCAC1	Cholangiocarcinoma cell	Promotes inflammatory stimulation	Xu et al. (2021)

EVs secreted by cancer cells are reported to contain a higher quantity of DNA fragments compared to those from normal cells. Notably, tumor extracellular vesicles harbor DNA reflecting the tumor’s genetic state, including the amplification of oncogenic gene c-myc (Balaj et al., 2011). These EVs are capable of transferring DNA to target cells, where the presence of double-stranded DNA (dsDNA) representing genomic DNA has been detected (Waldenström et al., 2012). Furthermore, detecting mutations within exosomal DNA has displayed substantial promise as a circulating diagnostic biomarker for cancer within clinical environments (Thakur et al., 2014).

## 3 Extracellular vesicles influence and shape tumor microenvironment by secreting cytokines

Interactions among the diverse cells within the tumor microenvironment are primarily mediated by soluble molecules. The tumor microenvironment (TME) can be likened to a persistent site of inflammation, characterized by a plethora of infiltrating and endogenous cells responsible for the synthesis and secretion of cytokines, chemokines, and growth factors, including but not limited to TNF-α, MMP-9, IL-6, and VEGF. These molecules possess the capability to orchestrate and modulate inflammatory responses (Komi and Redegeld, 2020). Recent research indicates that EVs from CCA cells trigger the release of pro-inflammatory cytokines and chemokines, such as IL-6, CXCL1, and CCL2, from cells in the TME (Figure 2). In reaction to this sequence of events, it promotes the proliferation of CCA cells via activation of the IL-6/STAT3 signaling pathway (Haga et al., 2015). The release of IL-6 by mesenchymal stem cells (MSCs) leads to an upsurge in the production of endothelin-1 by tumor cells, which triggers the activation of Akt and ERK pathways in endothelial cells, thereby amplifying their recruitment to the tumor site and bolstering the angiogenic processes (Huang et al., 2013).

### 3.1 IL-6 as the significant cytokine in the TME of CCA

IL-6 assumes a pivotal role in mediating communication between tumor epithelial cells and the established TME. Its expression in response to tumor cell-derived EVs is strongly associated with tumor growth. IL-6 holds a particularly crucial position in the initial phases of malignancy development within the biliary epithelium. In instances of CCA associated with oncogenic risk factors like trematode infection, cholestasis, primary sclerosing cholangitis (PSC), or biliary atresia, the underlying inflammatory condition of the liver can stimulate heightened IL-6 secretion (Chaiyadet et al., 2015; Liu et al., 2015). Other studies have demonstrated that enhanced telomerase activity, triggered by IL-6 incentive, can inhibit cellular senescence in malignant cholangiocytes, thereby promoting CCA growth (Yamada et al., 2015). IL-6 in promoting cholangiocarcinoma growth has been acknowledged (Braconi et al., 2010).

### 3.2 Chemokines represented by CXCL1 and CCL2

On the other hand, factors such as CXCL1 do not appear to influence cell proliferation significantly. There is speculation that these factors may exert additional effects that have the potential to influence tumor progression or metastasis. CXCL1 is involved in processes such as angiogenesis, inflammation, tissue repair, and oncogenesis (Haghnegahdar et al., 2000). Recent research has revealed that CXCL1, a CXCR2 ligand, significantly reduces the growth and mobility potential of CCA cells. Studies indicate a negative correlation exists between the expression of CXCL1 and the occurrence of distant metastasis, suggesting CXCL1’s role as a CCA progression suppressor. Additionally, CXCL1-CXCR2 signaling induces cancer cell senescence, triggering anti-proliferative responses and apoptosis. These mechanisms contribute to CXCL1’s tumor-suppressive effect, highlighting its potential in CCA progression. Overall, The CXCL1-CXCR2 axis could potentially exert a tumor-inhibitory function in the progression of CCA (Yamamoto et al., 2022). Conversely, monocyte chemoattractant protein-1 (MCP1/CCL2), a known target chemokine of FAK, serves as a robust catalyst for the invasion and migration of cancer cells (Dwyer et al., 2021). Currently, there is limited research on chemokines’ role in bile duct cancer, mainly due to the prominence of CCL2 in this process. Cytokines and growth factors transported by EVs in the TME of CCA mentioned in this article is summarized in Tables 4, 5.

**TABLE 4:** Cytokines of extracellular vesicles specifically expressed in the tumor microenvironment.

Name	Source	Function	References
IL-1β	Cholangiocarcinoma cell, TAM, DC, PLT, CAF, MSC	Promote inflammatory stimulation	Ponte et al. (2007)
IL-4	Cholangiocarcinoma cell, TAM	Promote inflammatory stimulation	Raggi et al. (2017)
IL-6	Cholangiocarcinoma cell, MSC, CAF, TAM, DC, Mast cell	Promotes inflammatory stimulation, angiogenesis, supports epithelial-mesenchymal transition (EMT)	Liu et al. (2015)
IL-8/CXCL8	Cholangiocarcinoma cell, MSC	Promote inflammatory stimulation	Ponte et al. (2007)
IL-10	Cholangiocarcinoma cell, TAM, CCA, Mast cell, CAF, MSC, DC	Promotes inflammatory stimulation and supports epithelial-mesenchymal transition (EMT)	Raggi et al. (2017)
IL-11	TAM	Promotes inflammatory stimulation	Zhou et al. (2021)
IL-12	Cholangiocarcinoma cell, DC, MSC	Promotes inflammatory stimulation	Baj-Krzyworzeka et al. (2007)
IL-13	Cholangiocarcinoma cell, TAM, CAF	Promotes inflammatory stimulation	Raggi et al. (2017)
CCL2/MCP-1	Cholangiocarcinoma cell, CAF, MSC, TAM	Promotes inflammatory stimulation	Ponte et al. (2007)
CCL5/RANTES	Cholangiocarcinoma cell, Activated T cells, MSC	Promote tumor invasion, inflammatory stimulation	Chen et al. (2011)
CCL17	TAM	Indirect immune effect	Colombo and Mantovani (2005)
CCL22	TAM	Indirect immune effect	Colombo and Mantovani (2005)
CXCL1	Cholangiocarcinoma cell, MSC	Promote tumorigenesis, inflammatory stimulation, angiogenesis, regulate tumor growth or spread	Huang et al. (2013)
CXCL9	CAF	Recruit tumor infiltrating NK cells	
CXCL12/SDF-1	Cholangiocarcinoma cell, CAF	Promote inflammatory stimulation, inhibit apoptosis, promote tumor invasion	Ponte et al. (2007)
TNF- α	TAM, Cholangiocarcinoma cell, MSC, DC, Mast cell	Stimulate inflammation, promote tumorigenesis, support epithelial-mesenchymal transition (EMT) and inhibit anti-tumor immune response	Ponte et al. (2007)
TGF-β	Cholangiocarcinoma cell, MSC	Promote angiogenesis and support epithelial-mesenchymal transition (EMT)	Techasen et al. (2012)
IFN-γ	Cholangiocarcinoma cell, MSC	Inhibition of anti-tumor immune response	Melzer et al. (2016)
PGE 2	Cholangiocarcinoma cell, MSC, Mast cell	Inhibit the anti-tumor immune response and enhance the malignant degree of tumor	Melzer et al. (2016)
CSF-1	Cholangiocarcinoma cell, TAM, MSC	Promotes inflammatory stimulation	Mitchem et al. (2013)
IDO	Cholangiocarcinoma cell, CAF, MSC	Inhibit anti-tumor immune responses and promote inflammatory stimuli	Cheng et al. (2016)

**TABLE 5:** Growth factors of extracellular vesicles specifically expressed in the tumor microenvironment.

Name	Source	Function	References
TGF	Cholangiocarcinoma cell, MSC	Promote angiogenesis and support epithelial-mesenchymal transition (EMT)	Melzer et al. (2016)
EGF	Cholangiocarcinoma cell, CAF	Regulate tumor growth, enhance tumor malignancy, promote tumor infiltration and invasion, and stimulate inflammation	Ponte et al. (2007)
PDGF	PLT, Cholangiocarcinoma cell, MSC, CAF	Promote inflammation and angiogenesis	Ponte et al. (2007)
PDGF-BB	CAF	Inhibition of anti-tumor immune response	Cadamuro et al. (2013)
PDGF-D	Cholangiocarcinoma cell	Promote the occurrence of tumor and regulate the growth of tumor	Cadamuro et al. (2019)
VEGF	MSC, CAF, TAM, MC, PLT, Cholangiocarcinoma cell	Promote angiogenesis and support epithelial-mesenchymal transition (EMT)	Melzer et al. (2016)
FGF	PLT, Cholangiocarcinoma cell, CAF	Promote tumor development and angiogenesis	Taraboletti et al. (2006)
HB-EGF	CAF	Promote tumor infiltration and invasion	Clapéron et al. (2013)

### 3.3 Multiple cells secrete and induce growth factors

In the TME of CCA, multiple cells, including tumor-associated macrophages (TAMs), cancer-associated fibroblasts (CAFs), and cancer cells, have the capability to secrete and stimulate the production of growth factors. Fibroblast activation in CCA is primarily driven by factors such as transforming growth factor-β (TGF-β), fibroblast growth factor (FGF), and platelet-derived growth factor (PDGF), which are secreted by TAMs and CCA cells. When activated, CAFs secrete various growth factors, including PDGF, heparin-binding EGF-like growth factor (HB-EGF), and vascular endothelial growth factor (VEGF), all of which play crucial roles in promoting cancer progression. PDGFs, particularly PDGF-D produced by CCA cells, function in a paracrine manner and exhibit proto-oncogenic effects. PDGF-D is instrumental in the recruitment and activation of CAFs by binding to PDGFRβ on their surfaces (Cadamuro et al., 2019). Growth factors transported and released by EVs are implicated in the advancement of CCA by promoting tumor growth, local invasion, migration, as well as new blood and lymph vessel formation, and the metastatic spread of tumor cells.

## 4 Extracellular vesicles and mesenchymal stem cells

MSCs within the TME exhibit the capacity to transform into various cell types and engage with cancer cells, thereby advancing tumor progression and metastasis. Multiple studies have elucidated the pro-tumorigenic influences of MSCs, foremost among them being (Ⅰ) the instigation of angiogenesis (Huang et al., 2013), (Ⅱ) drug resistance (Balakrishnan et al., 2010), (Ⅲ) immune response evasion (Poggi and Giuliani, 2016), (Ⅳ) stimulating EMT (Mele et al., 2014), and (Ⅴ) promoting metastasis (McAndrews et al., 2015). The interplay between MSCs and tumor cells initiates distinct reactions in both cell types, facilitated through direct and indirect connections. Indirect interactions are typified by the release of various molecules responsible for dampening immune responses, including TNF-α, IFN-γ, PGE2, and IL-6, IL-8, VEGF, PDGF, and TGF related to the process of angiogenesis (Melzer et al., 2016), as mentioned above, these cytokines, chemokines are delivered by tumor microenvironment EVs. Notably, MSC-derived extracellular vesicles (EVs) have also been found to facilitate immunosuppression, promote M2 macrophage polarization, and induce Treg cell formation (Zhang et al., 2014).

TME produce inflammatory reactions to recruit MSCs (Spaeth et al., 2008). Tumors secrete a variety of chemokines and cytokines, which have been demonstrated to engage with receptors on MSCs, including stromal cell-derived factor-1(SDF-1), epidermal growth factor (EGF), PDGF, monocyte chemotactic protein-1 (MCP-1), IL-8, and IL-1β, and TNF- α. These has been proven to stimulate the orientation of MSCs toward the tumor niche (Ponte et al., 2007). MSCs are recruited in reaction to inflammation with a specific attraction or affinity (Kerkelä et al., 2013). After implantation into the inflammation site, MSCs can engage with neighboring cells through either direct physical contact or through paracrine signaling effects (Zanotti et al., 2013). In the presence of chronic damage or inflammation, MSCs are readily recruited into the biliary tract, fostering bile duct cancer development (Braconi and Patel, 2010). This understanding paves the way to formulate approaches designed to inhibit the onset and advancement of CCA.

CCA induces MSCs via EVs, impacting the TME and promoting tumor growth. CCA releases EVs, like exosomes, taken up by MSCs, leading to increased mRNA transcription and secretion of CXCL-1, CCL2, and IL-6. Exposure to these EVs also triggers α-smooth muscle actin mRNA expression, selectively boosting myofibroblast biomarker α-smooth muscle actin and FAP in MSCs, enhancing their migratory abilities and possibly forming a cell-matrix transition. MSC-conditioned medium exposed to tumor-derived EVs activates STAT-3 phosphorylation, boosting tumor cell proliferation (Haga et al., 2015). Tumor cell-derived EVs elevate MSC fibroblast-like activity, promoting tumor matrix production through fibroblast differentiation. These EVs specifically influence the secretion of soluble substances, including IL-6, from MSCs, impacting tumor cell proliferation. These alterations in the release of cytokines and the differentiation of fibroblasts play a role in the promotion of tumor cell proliferation and the formation of the CCA stroma (Yang et al., 2015). MSCs are central to promoting cholangiocarcinoma initiation and progression by interacting with diverse TME cells, offering novel insights for both treatment and prevention.

## 5 Extracellular vesicles and cancer-associated fibroblasts

Cancer-associated fibroblasts (CAFs) are integral to shaping the tumor microenvironment, fueling tumor invasion, proliferation, and metastasis, thus fostering malignant tumor progression (De Wever et al., 2014). Recent insights emphasize the function of exosomes in facilitating communication between CAFs and cancer cells (Yang et al., 2017). CAFs secrete growth factors, chemokines, pro-inflammatory mediators, matrix metalloproteinases (MMPs), and extracellular matrix (ECM) constituents, orchestrating the expansion of tumors, the promotion of angiogenesis, and the attraction of bone marrow-derived cells to primary tumor sites, ultimately facilitating metastasis (Fullár et al., 2012; Deng et al., 2017). Inappropriate or abnormal activation of signaling pathways, such as IL-6/STAT3, FGF-2/FGFR1, NF-κB, and TGF-β1/SMAD, distinguishes CAFs from normal fibroblasts (NFs). CAFs release specific exosomes, which are taken up by cancer cells, facilitating the transfer of various bioactive substances. Reciprocally, cancer cell-derived EVs promote CAFs conversion. This complex interaction is a contributing factor to the advancement and development of the tumor.

CAFs are the CCA matrix primary components, crucial in mediating its growth and progression. In CCA, their abundance correlates with tumor expansion and poorer survival (Banales et al., 2020). CAFs, through the secretion of immunomodulatory factors, exert significant control over the TME. They regulate the innate immune response by promoting the M2 polarization of macrophages and inhibiting the activation of natural killer (NK) cells. Furthermore, CAFs stimulate the generation of regulatory T cells and T helper 2 cells while hindering the activity of dendritic cells and cytotoxic T cells, influencing adaptive immunity dynamics (Banales et al., 2016). In iCCA, CAFs exhibit heightened expression of IL-6, and the IL-6/IL-6R axis is heightened in both CAFs and tumor cells. This pro-inflammatory milieu activates the IL-6/STAT3 axis, prompting CAFs to release increased amounts of IL-6 and the immunosuppressive enzyme indoleamine 2,3-dioxygenase (Cheng et al., 2016; Zheng et al., 2016). Consequently, CAFs fulfill a critical function in inducing dendritic cells (DCs) to adopt a regulatory state, reducing antigen-presenting capabilities, impairing the recruitment and activation of tumor-infiltrating lymphocytes (TILs), and enhancing the inhibition of myeloid-derived suppressor cells (MDSCs) through the fibroblast activation protein (FAP) and STAT3 axis. This intricate interaction participates in the formation of an inflammatory TME (Yang et al., 2016).

Several chemokines primarily secreted by CAFs assume pivotal roles in the interaction between the tumor and the immune microenvironment in CCA. CCL2 lures MDSCs to the TME, promoting CCA expansion (Lin et al., 2019). CXCL9 governs the attraction of tumor-infiltrating NK cells in CCA. Elevated CXCL9 levels correlates with improved overall survival after surgical resection (Fukuda et al., 2020). These discoveries indicate that targeting chemokines in the TME of CCA holds promise as a therapeutic approach to impede CCA growth and modulate the immune response.

As mentioned above, CAFs actively release a diverse repertoire of growth factors. PDGF-BB, secreted by myofibroblasts, it thwarts apoptosis triggered by TNF-α in CCA cells by activating PDGF receptor β (PDGFR β) in these cells (Fingas et al., 2011). HB-EGF, which is released by CAFs, functions as a ligand for EGFR. This activation of EGFR in CCA cells results in the stimulation of their *in vitro* migration and invasion. The use of neutralizing antibodies to block HB-EGF can effectively hinder the progression of CCA (Clapéron et al., 2013). Furthermore, after being stimulated by PDGF-D from cholangiocytes within the tumor, CAFs release VEGF-A and VEGF-C. These factors then attract and orchestrate the arrangement of lymphatic endothelial cells into vascular formations that are conducive to the intravasation of tumor cells. Navitoclax inhibits metastatic spread of the tumor in live organisms by inhibiting the release of VEGF-A/C by activated CAFs in CCA (Cadamuro et al., 2019).

In the carcinoma microenvironment, fibroblasts often adopt an altered phenotype, marked by increased expression of α-smooth muscle actin (α-SMA) and fibroblast activation protein (FAP). CAFs notably express α-SMA (Vaquero et al., 2020), and elevated α-SMA levels within the tumor stroma are linked to enhanced survival among patients diagnosed with CCA (Zhang et al., 2017; Itou et al., 2019). Moreover, periostin, an extracellular matrix protein synthesized by α-SMA-positive CAFs in iCCA, exhibits elevated expression compared to control tissues (Sirica et al., 2014). Increased periostin levels act as an indicator of malignant progression in CCA and are associated with a less favorable prognosis for patients (Manzanares et al., 2018).

Tumor cells exploit miRNAs carried by EVs as signaling agents that promote the establishment and activation of CAFs, subsequently influencing tumor cell behavior. Dysregulation of miRNAs is intimately connected to the activation and development of CAFs, impacting their tumor-supporting capabilities both within a controlled laboratory setting and within living organisms. MiRNAs within CAF-derived EVs can modulate the migration, infiltration, and metastasis of tumor cells, as well as the induction of drug resistance and determine aggressive cancer phenotypes. For instance, CAFs secrete exosomes enriched with miR-493-5p, promoting tumor progression. MiR-493-5p’s target expression, cocaine-amphetamine-regulated transcriptional precursor peptides, significantly correlates with intrahepatic cholangiocarcinoma (iCCA) prognosis (Toshida et al., 2023). Elevated miR-9-5p in EVs from iCCA cells induces IL-6 expression in CAFs, upregulates EZH2 in iCCA cells, and enhances tumor malignancy (Zhang et al., 2020). Conversely, downregulated miR-30e in human CCA cells can be partially restored by treating them with miR-30e-enriched EVs, leading to attenuated cell invasion and migration (Ota et al., 2018; Trifylli et al., 2023).

As vital constituents of the TME in CCA, CAFs, MSCs, and tumor cells utilize EVs as conduits for the transportation of diverse soluble molecules, including cytokines, chemokines, and growth factors (Figure 2). This intricate interplay serves as an imperative cog in the machinery of tumor progression. Through the exchange of these substances, the triad of cell types mutually propel and invigorate each other, instigating a cascade of events that promote inflammatory stimulation, angiogenesis, and the relentless expansion of tumorous growth. The complex interaction between CAFs and CCA cells, as well as immune cells, and MSCs continues to evolve, offering potential therapeutic targets. Given CAFs’ pivotal role in CCA progression, targeting them is considered a promising therapeutic strategy for CCA.

## 6 Immune cells and tumor cell-derived EVs act as modulators of tumor immunology

### 6.1 Macrophage

Tumor-associated macrophages (TAMs) are a diverse cell group within the TME, crucial for supporting CCA cell growth by releasing cytokines, chemokines, and growth factors, promoting angiogenesis, and suppressing specific immunity (Techasen et al., 2012; Raggi et al., 2017).

TAMs play a pivotal role in bridging the link in connecting the immune response to cancer by dampening adaptive immunity via cytokine secretion (Sica et al., 2008). Studies have revealed that TAMs release immunosuppressive cytokines, including IL-6 and IL-10, which hinder the protective functions of tumor-infiltrating lymphocytes (TILs), thus facilitating the process of epithelial-mesenchymal transition (EMT) and subsequent metastasis. Additionally, research has demonstrated the abundant secretion of OSM and IL-11, both belonging to the IL-6 cytokine family, by TAMs during inflammation and cancer. TAMs collaborate to advance iCCA progression through the OSM/IL-11/STAT3 signaling pathway (Zhou et al., 2021). Simultaneously, M2-polarized macrophages activated by iCCA support tumor expansion and invasiveness by means of EMT induced through the IL-10/STAT3 pathway. This provides a potential focus for therapeutic interventions aimed at iCCA (Yuan et al., 2020). TAMs exert their immunosuppressive effects indirectly by releasing chemokines that selectively attract T cell subsets deficient in cytotoxic capabilities. For instance, TAMs abundantly express chemokines like CCL17 and CCL22 that engage with the CCR4 receptor, primarily located on Th2 cells and Tregs, two T cell subsets with restricted anti-tumor capacities (Colombo and Mantovani, 2005). Moreover, chemokines within the tumor microenvironment have been demonstrated to enhance the recruitment of macrophages. The polarization of macrophages toward a tumor-promoting M2 state and increased TAM infiltration in CCA have been associated with poor prognosis and metastasis in CCA (Thanee et al., 2015; Kaneda et al., 2016). EVs derived from CAFs and tumor cells carrying CCL2 further support macrophage recruitment from circulating monocytes (Mitchem et al., 2013). Chemokines (e.g., CCL5, CCL7, CCL8, CXCL12) or cytokines such as VEGF, PDGF), and macrophage colony-stimulating factor (M-CSF) have also been confirmed to entice peripheral blood monocytes to the TME (Mantovani et al., 2006).

In the tumor stroma, many products released by macrophages can directly stimulate tumor cell proliferation, mobility, and metastatic spread. These products include epidermal growth factor (EGF), members of the FGF family, TGF-β, VEGF, PDGF, as well as various chemokines and cytokines. TAMs may play a role in promoting tumor advancement by aiding in the development of the stroma and the process of angiogenesis. They achieve this by releasing PDGF, which works in tandem with TGF-β produced by cancer cells. The accumulation of TAMs is associated with heightened angiogenesis and the secretion of angiogenic factors such as VEGF and PDGF. Additionally, research conducted in both controlled laboratory settings and living organisms has demonstrated that TAMs have the capacity to induce the expression of PD-L1 in both mouse and human cancer cells through the secretion of EGF (González and Falcón-Pérez, 2015).

EVs released by TAMs transport miRNAs to cells in the TME, contributing to pro-tumoral processes, including promoting cancer cell proliferation, migration, invasion, regulating immune cells to facilitate immune escape, and enhancing cancer cell resistance to anti-cancer drugs (Cocks et al., 2022). Notably, exosomal miR-183-5p downregulates the phosphatase and tension homolog (PTEN) expression, leading to heightened levels of phosphorylated AKT and PD-L1 expression in macrophages. Clinical data has indicated that elevated plasma exosomal miR-183-5p levels are associated with an unfavorable prognosis in iCCA patients following radical resection. Thus, exosomal miR-183-5p holds potential as a biomarker for predicting iCCA progression and as a target for developing therapeutic strategies to address immune tolerance features in iCCA (Shu et al., 2023).

### 6.2 Natural killer (NK) cell

As a crucial cell in the innate immune system, NK cells maintain homeostasis, resist viral incursions and thwart the survival or dissemination of malignantly altered cells. Preclinical investigations have indicated that the absence of NK cells or impaired NK cell function is linked to the advancement of tumors (Peng et al., 2017; Jun et al., 2019). The equilibrium between activation and inhibitory signals plays a crucial role in determining the activation status of NK cells. NK cells can be triggered into activation by HSP70 present in EVs, either in its soluble form or as a membrane-bound protein (Kim et al., 2006). The release of HSP70-containing EVs from tumor cells activates NK cells, which can reduce tumor growth by recognizing stress-induced NKG2D ligands on malignant cells (Elsner et al., 2010). The natural killer group 2D (NKG2D) is an activating receptor on NK cells, and it is responsible for targeting and eliminating tumor cells by binding to its ligand, NKG2D ligand (NKG2DL). Disruption or impairment of the NKG2D/NKG2DL axis contributes to the ability of tumors to evade detection by the immune system. Variations in the NKG2D receptor identified in individuals with PSC have been documented to heighten their vulnerability to CCA (Melum et al., 2008; Wadsworth et al., 2019). Additionally, elevated levels of NKG2D ligands in human CCA were linked to better disease-free and overall survival among patients (Tsukagoshi et al., 2016).

Human NK cells themselves constitutively release EVs. NK cell-derived EVs exhibit cytotoxicity to tumor cells and activated immune cells (Clayton et al., 2008; Lugini et al., 2012). Summarily, both EV derived from NK cells and EV released by stress cells or tumor cells can contribute to the regulation of the immune system by stimulating NK cells.

### 6.3 Dendritic cell (DC)

Within the TME, DCs occupy a pivotal role, serving as a linchpin in initiating and orchestrating both the innate and adaptive immune reactions. Antigens carried to DCs through EVs have the capability to trigger antigen-specific T cell reactions. The EV-delivered antigens released by tumor cells can suppress immune responses against the tumor, such as hindering the activation of T cells or DCs (Fabris et al., 2021). In CCA, the penetration of fully developed CD83^+^DCs is associated with the accumulation of CD4^+^/CD8^+^T cells in the surrounding area of the tumor. The existence of CD83^+^DCs cells was also linked to enhanced patient prognoses. On the contrary, immature CD1a DCs in the central part of tumors was correlated with the lack of CD4^+^/CD8^+^ T cells (Takagi et al., 2004). There are also studies indicating that FcεRI^+^ (a high-affinity immunoglobulin E receptor) monocytes and DCs in the bloodstream of individuals diagnosed with CCA were significantly decreased. These results suggest that DCs in CCA are impaired in their function and are unable to restrain the advancement of the tumor (Martín-Sierra et al., 2019).

EVs derived from tumors can modulate the immune responses of the host through a range of pathways, which includes promoting pro-inflammatory effects (Wang et al., 2022). Tumor-derived EVs induce monocytes to release pro-inflammatory cytokines. Research has shown that monocyte stimulated by EVs originating from cancer cells heightened the human leukocyte antigen DR (HLA-DR) expression, the production of reactive oxygen intermediates, mRNA accumulation, and the release of TNF, IL-10, IL-12 (Baj-Krzyworzeka et al., 2007). Crucially, EVs play a pivotal role in inducing liver inflammation, a hallmark of nearly all liver conditions, encompassing acute liver damage, chronic viral hepatitis, hepatocellular carcinoma (HCC), and cholangiopathy (Tkach et al., 2022). Various Clinical trials have seen the development of vaccines targeting DCs to augment cancer immunotherapy. Prior research has highlighted the feasibility of utilizing tumor-derived exosomes (Texs) as cell-free carriers for *in situ* DC activation in the TME (Huang et al., 2022). This approach, incorporating Tex-based delivery of stimulants and adjustable adjuvants to malignant cells may present a potential approach for treating CCA.

### 6.4 Mast cell

Mast cells have a critical function in innate immunity and the control of adaptive immunity through the secretion of various immunomodulatory mediators (Rodewald and Feyerabend, 2012). Mast cell-derived EVs contain immunomodulatory proteins, including MHC II, leukocyte function-associated antigen 1 (LFA-1), intercellular cell adhesion molecule-1 (ICAM-1), Heat Shock Proteins (HSP), and high-affinity IgE receptors (Carroll-Portillo et al., 2012). These EVs can home in on additional mast cells, provoke the maturation of DCs, transport antigens for cross-presentation, and activate B and T cells (Skokos et al., 2003). Within the TME, mast cells interact by engaging in direct cell-to-cell interactions with infiltrated immune cells, tumor cells, and the extracellular matrix (ECM), or through the release of a wide array of mediators that are capable of reshaping the TME. Mast cells play an active role in promoting angiogenesis by releasing both traditional proangiogenic factors such as VEGF, FGF-2, PDGF, and IL-6, and non-traditional proangiogenic factors, primarily proteases like tryptase and chymase. Moreover, mast cells facilitate tumor invasiveness by secreting a diverse array of matrix metalloproteinases (MMPs).

The process of interaction between mast cells and the TME of CCA can be initially observed in bile. Mast cell can infiltrate the liver during cholestasis and cause biliary damage. Within the model of bile duct ligation, increased Mast cells were observed around the damaged bile duct and increased biliary hyperplasia, liver injury, and fibrosis induced by bile duct ligation (Meadows et al., 2019). Prior investigations have likewise demonstrated that Mast cells may be involved in CCA progression by releasing histamine (HA) in CCA (Kennedy et al., 2018). Bile exosomal miR-182/183-5p is released by CCA cells targets the hydroxyprostaglandindehydrogenase (HPGD) in CCA cells and Mast cells and increases the production of Prostaglandin E2 (stimulates PTGER1), thereby promoting the proliferation, invasion, EMT of CCA. miR-182/183-5p also promotes angiogenesis by releasing VEGF-A expression to promote Mast cell release of VEGF-A. The study demonstrated for the first time that Mast cells constitute the predominant cell type within HPGD and also the target cells of bile exosomal miR-182/183-5p (Shu et al., 2023). Mast cells are key factors in the bile interaction of CCA, promoting CCA progression through the release of PGE2 and VEGF-A. The significant role of mast cells in the advancement, angiogenesis, and fibrogenesis of CCA suggests the possibility of treating CCA through the local administration of Mast cell stabilizers or anti-angiogenic drugs in the bile duct.

### 6.5 Tumor-infiltrating lymphocytes (TILs)

Tumor-infiltrating lymphocytes (TILs) consist of B lymphocytes, cytotoxic T cells (CD8^+^T), and T helper cells (CD4^+^T). The cellular makeup and molecular profiles of TILs reconfigure the CCA microenvironment, impacting the immune surveillance or evasion of cancer. Upon activation, T cells can generate immunomodulatory EVs carrying MHC, TCR, APO2 ligands, Fas ligand (FasL), and NKG2D ligands. These EVs have been shown to inhibit NK cytotoxicity (Alonso et al., 2011; Hedlund et al., 2011), block T cell stimulation (Busch et al., 2008), promote T cell apoptosis (Monleón et al., 2001), and diminish the stimulatory ability of antigen-presenting cells to activate T cells, thereby contributing to the suppression of the immune response (Xie et al., 2010). Research has demonstrated that extracellular matrix protein degradation via MMP-9 mediation may be heightened by CD8^+^ T cell-derived exosomes featuring membrane-bound FasL, consequently intensifying the invasive and metastatic potential of Fas^+^ tumor cells (Cai et al., 2012).

Although T cells constitutively release EV, T cell receptor (TCR) triggering and intracellular calcium stimulation increase EV secretion (Blanchard et al., 2002). In addition to immunosuppressive effects, EVs originating from T cells have been associated with the induction of T cell proliferation through a mechanism that relies on CCL5 (Wahlgren et al., 2012) and enhancement of immunogenicity is achieved by selectively modulating gene regulation within antigen-presenting cells (APCs) (Spaeth et al., 2009). The immune synapse interactions occur both at the interface of CD8^+^ cytotoxic T cells and tumor cells, as well as at the crossroads of T cells and APCs, is an efficient mechanism for the transfer of EVs (Mittelbrunn and Sánchez-Madrid, 2012). It was demonstrated that BMI1 suppresses CD8^+^ T cell chemokine recruitment by facilitating inhibitory H2A ubiquitination in CCA cells. BMI 1 is an indicator associated with an unfavorable prognosis for CCA. The unique exosomes containing BMI 1 promote the multiplication and metastasis of CCA via an autocrine/paracrine signaling pathway (Liu et al., 2022).

EVs secreted by various effector T cell subsets, including Th1, Th2, and Treg, exhibit unique miRNA profiles. Specific miRNAs in EVs derived from Treg cells inhibit pathogenic Th1 cells and inflammation (Seo et al., 2018). FoxP3 is a protein crucial for the formation and role of Tregs, and its downregulation in CCA cells leads to the reduction of TGF-β, thereby improving the survival of effector T cells (Ma et al., 2015). Similarly, the overexpression of FoxP3 within pancreatic ductal adenocarcinoma (PDAC) cells leads to the increased transcription of PD-L1 and the recruitment of Tregs, ultimately augmenting immune evasion by the tumor (Wang et al., 2020).

Adoptive cell therapy (ACT) is a form of cancer immunotherapy that harnesses the patient’s own immune cells to seek out and eliminate malignant cells. Initial discoveries showed that T cells were responsible for graft-versus-tumor responses. In the case of Tumor-Infiltrating Lymphocyte (TIL) ACT, TILs are extracted from surgically removed tumor tissue, expanded and enhanced outside the body, and subsequently reintroduced into the patient as therapeutic agents. ACT using TILs has displayed the capacity to induce significant tumor regression in various cancer types, including cholangiocarcinoma. In cancer immunotherapy, both CD8^+^ and CD4^+^ T cells play a role in combating tumors. Nonetheless, the field has predominantly focused on comprehending the anti-tumor cytotoxicity mediated by CD4^+^ T cells (Kumar et al., 2021). For instance, in TIL cultures from a cholangiocarcinoma patient, MHC class 2 (MHCII) antigen HLA-DQ O6-restricted CD4^+^ T cells that recognize the ERBB2IP mutation were identified (Tran et al., 2014). Several strategies are presently under clinical development for the identification of tumor-associated antigens and the creation of personalized Adoptive Cell Therapy (ACT) products to enhance the effectiveness of tumor control. One of the most advanced methods for personalizing Tumor-Infiltrating Lymphocyte (TIL) ACT is the identification and expansion of TILs that possess T cell receptors (TCRs) specifically tailored to target tumor neoantigens. Extracellular vesicles have the potential to act as antigen-presenting tools and could catalyze further progress in the development of ACT.

## 7 Extracellular vesicles and angiogenesis

The initiation and sustenance of tumor neovascularization are governed by a intricate network of interactions (Bikfalvi, 2003). EVs are implicated in various aspects of vascular regulation in malignancy. Tumor- and platelet-derived EVs are rich sources of angiogenic growth factors (VEGF, FGF) (Taraboletti et al., 2006) and pro-inflammatory cytokines [IL1β (Bianco et al., 2009)]. Growth factors and their receptors or adhesion molecules could expand neovascularization through synergistic effects with extracellular matrix (Carmeliet et al., 2009; Hong et al., 2009). These may contribute directly or indirectly to a pro-angiogenic intratumoral environment, possibly through several processes (Dolo et al., 2005; Ratajczak et al., 2006), including 1) cell-to-cell transfer of pro-angiogenic substances, 2) extracellular release of the pro-angiogenic contents of EV, or by 3) inducing pro-angiogenic alterations in gene expression following the interaction of vascular cells with EVs (Mostefai et al., 2008). For example, endothelial progenitor cells release EVs carrying mRNA that can be conveyed to local endothelial cells, inducing angiogenesis activation (Deregibus et al., 2007). Endothelial cells can likewise react to the transfer of mRNA from tumor cells or the uptake of EVs carrying the active EGFR oncoprotein (Al-Nedawi et al., 2009). In the latter scenario, endothelial cells display EGFR positivity both *in vivo* and *in vitro*, leading to the initiation of endogenous or autocrine angiogenic activity, such as VEGF production.

The primary role of VEGFs as specific growth factors for vascular endothelial cells is the induction of angiogenesis. VEGF is markedly upregulated in CCA and correlated with unfavorable patient outcomes (Vaeteewoottacharn et al., 2016). Platelets release EVs containing VEGF (Kim et al., 2004). Furthermore, it has been demonstrated that this factor is found within tumor-derived EVs and is exclusively released from EVs in a biologically active form under the acidic pH conditions of the TME (Taraboletti et al., 2006). VEGF-D modulates the function of stromal cells and facilitates tumor cell metastasis via lymphatic dissemination (Stacker et al., 2001). VEGF-C secreted by CAFs can enhance lymphatic endothelial cells’ (LECs) permeability, hence, promoting lymphoid infiltration and metastasis in CCA (Cadamuro et al., 2019). Single-cell transcriptome analysis of tumors from HCC patients, including 9 iCCA patients, revealed that VEGF is indispensable in intratumoral diversity (Ma et al., 2019). The results indicate that VEGF, induced by Hypoxia-Inducible Factor 1-α (HIF1α), orchestrates the manipulation of tumor endothelial cells, CAFs and TAMs, promoting tumor advancement. These findings suggest that blocking VEGF could potentially impede the progression and metastasis of CCA. Regorafenib, an oral multi-kinase inhibitor that targets VEGFR2, demonstrated a significant inhibition of CCA growth both *in vitro* and *in vivo* (Yeh et al., 2017).

Coordinated changes in promoting angiogenesis can be induced in multiple cells. One way to achieve this effect may be through the intercellular exchange of EVs (Al-Nedawi et al., 2008). When circular RNA circ-CCAC1, originating from EVs derived from CCA, was introduced into endothelial monolayers, the integrity of the endothelial barrier was disrupted and induced angiogenesis, leading to the tumorigenesis and metastasis of CCA tumors (Xu et al., 2021). Circular RNA circ-CCAC1 plays a pivotal role in the tumorigenesis and metastasis of CCA and could potentially serve as a significant biomarker and therapeutic target for CCA (Trifylli et al., 2023).

## 8 Discussion

EVs play a crucial role in the growth and advancement of cancer, which encompasses the establishment of the tumor microenvironment, angiogenesis, and the stimulation of tumor growth and invasion. Disrupting the generation, emission, and absorption of EVs at various stages can be considered a viable therapeutic strategy for cancer. Among them, intervention in the production of ceramide is the most commonly employed approach to reduce the production of exosomes in tumor. Suppression of ceramide production using myriocin, a selective inhibitor of serine palmitoyltransferase, diminished the pro-inflammatory properties of EVs derived from iCCA, inhibition of neutral type II sphingomyelinase (nSMase2), which accountable for the synthesis of ceramide, and its inhibitor GW4869 can reduce exocrine secretion (Trajkovic et al., 2008; Singh et al., 2014) and make cancer cells sensitive to chemotherapy (Richards et al., 2017). Notably, GW4869 inhibited the migratory ability of CCA cells (Haga et al., 2015). Obstructing or eliminating cancer-generated EVs through apheresis utilizing specialized devices represents a promising therapeutic method. In the meantime, EVs are also a strong contender for delivering novel anticancer proteins, drugs, or cancer vaccines.

As an antigen carrier, EV provides a new method for exploring personalized immunotherapy for cholangiocarcinoma. Among them, immunotherapy is the most widely studied. Exosomes derived from AFP-expressing dendritic cells (DCs) (DEXAFP) induced specific and robust immune responses against the antigen. They caused significant tumor growth retardation and prolonged survival in HCC tumor mice induced by ectopic, *in situ*, or carcinogens. In hepatocellular carcinoma (HCC) mice subjected to DEXAFP treatment, the TME showed notable enhancements. This was evident through a substantial increase in the count of CD8^+^ T lymphocytes expressing IFN-γ, elevated levels of IFN-γ and IL-2, a reduction in the quantity of CD25^+^ Foxp3^+^ Treg cells, diminished levels of IL-10, and a decrease in the presence of transforming TGF-β at the tumor sites (Rao et al., 2016). DC-derived exosomes offer innovative avenues for the development of cancer immunotherapy vaccines (Lu et al., 2017). Apart from its involvement in EV-based therapy, what is contented in EVs can serve as an indicator of response to immunotherapy (Zuo et al., 2020). Therefore, the release of EVs from tumor cells or circulating exosomes presents a novel therapeutic target. Exosomes can serve as a vehicle for the targeted transport of medications, therapeutic compounds, or gene therapy materials to tumors.

Moreover, exosomal molecules can serve as biomarkers for the early detection and diagnosis of diseases, as well as for assessing prognosis and predicting therapeutic outcomes based on the molecular signatures of exosomes (Zhang and Grizzle, 2011). Vesicles displaying exosome-like traits have been successfully isolated from a range of bodily fluids, including blood (Caby et al., 2005), urine (Huebner et al., 2015), bile (Masyuk et al., 2010), malignant effusion (Andre et al., 2002). Recent research findings have indicated that miRNA-based panels exhibit specificity for CCA and are derived from miRNAs isolated from human biliary EVs, thus indicating the potential utility of biliary EVs as a diagnostic approach (Arbelaiz et al., 2017; Sirica et al., 2019; Lapitz et al., 2020). In summary, these investigations suggest that exosome proteins can be considered early diagnostic biomarkers rather than just proteins in the tumor itself. Currently, the most significant obstacle to the effective utilization of EVs as a diagnostic and prognostic tool lies in the considerable inter-individual variability in protein content within various bodily fluids, such as urine and blood. Additionally, the requirement for standardized procedures for preserving, purifying, and examining vesicles from diverse bodily fluids imposes notable constraints. This factor implies that the existing gold standard technique for EV isolation is not applicable in clinical contexts (González and Falcón-Pérez, 2015). Extensive research is still needed before some enter routine clinical practice.
